# An Innovative and Portable Multimodal Pain Relief Device for the Management of Neuropathic Low Back Pain - a Study from Kashmir (Southeast Asia)

**DOI:** 10.7759/cureus.661

**Published:** 2016-06-29

**Authors:** Shah Faisal Ahmad Tarfarosh, Baseer-ul-Rasool Lone, Mirza-Idrees-ul-Haq Beigh, Mushbiq Manzoor

**Affiliations:** 1 MBBS, Acharya Shri Chander College of Medical Sciences and Hospital, Jammu, J & K, India; 2 Surya Educational Charitable Trust, Shambubarier, Punjab Technical University; 3 MIET, University of Jammu; 4 MBBS, Sheri Kashmir Institute of Medical Sciences Medical College, Srinagar, India

**Keywords:** neuropathic low-back pain, wearable technology, biomedical engineering, health technology, adjuvant therapy, back pain, chronic pain, neuropathy, neurological disorders, interventional neurology

## Abstract

We developed a portable multimodal system with seven different mechanisms of pain relief incorporated into a lumbar belt called the Comfort-N-Harmony Belt (C&H belt).

Here, we describe the technical details of the system and also summarize the effects of this multimodal pain relieving technology as an adjuvant to analgesics versus analgesics alone, on the level of pain, improvement of psychological status, disability, and the quality of life in the patients with neuropathic low back pain (LBP).

We tracked the volunteers who were following up at a tertiary health care center for the complaints of neuropathic LBP of minimum three months duration and were on analgesics alone with no relief in the severity of the pain. Study group A (n = 45) consisted of volunteers with LBP on C&H belt therapy, along with the usually prescribed analgesic intake, and group B (n = 45) with LBP volunteers on analgesics, plus a similar looking but plain leather belt (placebo). For pain, the VAS (Visual Analogue Scale); for anxiety and depression, the (HADS) Hospital Anxiety-Depression Scale; for disability, the RMDQ (Roland Morris Disability Questionnaire); and for quality of life, (NHP) Nottingham-Health-Profile were used before and after the study period.

There were no significant differences in demographic variables between the groups (p < 0.05). After the study period of one month, VAS, RMDQ, NHP-pain, NHP-physical activity, and HADS scores in both groups were significantly improved compared to the pre-treatment scores (p < 0.05). Group A also showed significant improvements in the scores of NHP-energy level and NHP-social isolation (p < 0.05). The post-treatment scores did not significantly show any difference between the two groups (p > 0.05). However, in comparison of pre- and post-treatment scores, the pre-treatment score values of RMDQ, NHP-pain, NHP-physical activity, and NHP-social isolation were much higher in group A compared to the group B, but still these scores were, in a statistically significant manner, improved in group A compared to the group B after the study period was over (p < 0.05).

Multiple pain relieving mechanisms in a portable device-based system, when used along with analgesics, are effective in relieving pain, improving function and quality of life, and help in relieving the associated anxiety and depression in patients with chronic neuropathic LBP than the analgesics alone in the Kashmiri (Southeast Asian) population.

## Introduction

Chronic low back pain (LBP) consists of both the nociceptive and the neuropathic components. The neuropathic component appears to be under-recognized as well as undertreated. Not only is this neuropathic component challenging to manage, but also many patients with chronic LBP have been known to suffer from the type of pain, which is more or less refractory to existing treatments [1]. It has been estimated that between 5-10% of cases of mild but persistent lower backaches will develop into chronic LBP, which is responsible for the high treatment costs, absenteeism from work, and individual suffering. It is also claimed to be one of the topmost reasons for people of both developed as well as developing nations to seek health care services. The worst part is that the information about the prevalence of LBP and its associated factors are scattered in the literature [2]. Only less than 50% of these patients experience clinically meaningful analgesia with oral analgesic intake. Even the oral analgesics are associated with risks of numerous adverse effects. While NSAIDs are widely used for the management of LBP, these are unlikely to ameliorate the neuropathic component of this pain. Moreover, the data on the role of antidepressants and gabapentin or pregabalin for neuropathic pain are limited [1].

LBP is a major cause of functional limitations as well as disability. It has been found that the people suffering from back pain for longer periods have worse physical and mental health than people without back pain. Furthermore, those with LBP are three times more likely to have limited functional ability and a risk of over four times to experience serious psychological distress than people without LBP [3].

Low back pain is also known to be a common health problem arising from work with manual handling. This type of work (like farming) is very common in the developing nations of the Southeast Asian region [4-5].

A large number of treatment modalities have been employed for managing the chronic LBP. These include the medical treatment, massage, physical therapy, traction, manipulation, and the various therapeutic exercises. Among these modalities, the role of physical therapy and psychological treatments is still valued by doctors as well as patients with chronic neuropathic LBP [6].

Patients suffering from LBP resistant to prescribed analgesics have found the conventional medical treatments to be ineffective as well as unreliable for treating their pain. Because of this high level of dissatisfaction with conventional treatments for LBP, increasing numbers of people suffering from LBP are turning to complementary and alternative medicine (CAM) to find relief [7]. There is a considerable amount of scientific evidence that supports the use of CAM for improving back pain outcomes, with back pain being the most common condition for which patients turn to CAM [8-10].

However, it is definitely not our aim to label the non-conventional methods of pain relief to have an undue importance over the conventional allopathic systems. We believe that until the medical science comes up with a single most effective pain relief medication for this neuropathic component of LBP, we should use the non-conventional, yet proven, systems of pain relief as at least “adjuvants” to the current pharmacological intervention for pain relief. What is better than combining all those proven pain relief mechanisms (in one single device) that have a great deal of acceptance and compliance with the chronic LBP patients?

The main purpose of the present study was to evaluate and compare the effectiveness of medical therapy alone and medical therapy coupled with an effective multimodal physical therapy on the pain, psychological status, disability, and the quality of life of individuals with chronic LBP in Kashmiri (Southeast Asian) population.

## Materials and methods

The General Medical Council for Ethics and Research approved our study. No protocol number is required in India. Written informed consent was obtained from all volunteers/participants. No reference to the participant's identity was made at any stage during data analysis, in the media used, or in the body of the paper.

The study participants included Kashmiri volunteers with the chief complaints of pain in the lower back of at least three months duration following up at a tertiary health care center. The volunteers were classified into two groups in a random double-blind fashion: those who were scheduled for physical therapy (C&H belt), along with their usual prescribed analgesics, belonged to Group A (n = 45). Those in whom a similar but plain lumbar belt (having no scientifically proven pain relieving mechanisms) was used as a placebo, along with their usual prescribed analgesics, were kept in Group B (n = 45). The volunteers were clearly informed about the aim of our study. Then, the informed consent was obtained from the volunteers. Those who accepted to participate in the study were included. The sociodemographic characteristics of each of the volunteers were noted down.

Our exclusion criteria included: Inflammatory and rheumatic diseases, any local lumbar infection or a systemic infection, low back pathologies leading to the neurological defects in lower limbs, connective tissue diseases, tumors (benign or malignant) in the lumbar region, any history of spinal fractures in the recent past, and those participants with diagnosed somatoform disorders. Volunteers who met the exclusion criteria were not included in the study.

An overview of the different potential pain relief mechanisms that we used in this multimodal device-based system for pain relief is as follows:

a. Vibration by an effect called vibratory analgesia: We have fixed three vibrators in the device that can be controlled on the outside by control knobs for frequency and for activating one, two, or all three vibrators at a time.

b. A heating effect for pain relief: We have placed a therapeutic level heat generating heating pad, which has a control knob for adjusting the intensity of heat.

c. A cooling effect for pain relief: High-quality peppermint oil (tested for topical use) has been placed in one of the two roll-on bottles whose roller moves with the movement of the vibrators, releasing oil in little quantities over the affected part.

Note: The heating and cooling effects of the device were alternately used (each for 10 minutes duration).

d. Irritation of nerves: Counterirritants have been known to significantly reduce pain by various molecular mechanisms.

A high-quality camphor oil (tested for topical use) has been placed in one of the two roll-on bottles whose roller moves with the movement of the vibrators releasing oil in little quantities over the affected part.

e. Reducing the range of motion of the trunk and increasing its stiffness: This effect is exerted by this belt as it is strategically tightly fixed around the lower back and waist.

f. Endorphin release: Endorphins are chemical substances produced inside the body leading to pain relief.

We used another aromatic oil, dilute lemon oil (tested for topical use) with a pleasant fragrance, as diluent for camphor and peppermint (which themselves are categorized as aromatic oils) to release the body’s own endorphins (aromatherapy).

g. Magnetic therapy: Some studies have demonstrated a little degree of analgesia, although the absence of analgesic effect has not been clearly proven yet.

We have placed numerous magnets scattered in the belt, which do not interfere with the working of the device and at the same time perform their own work of potential pain relief.

**Figure 1:**
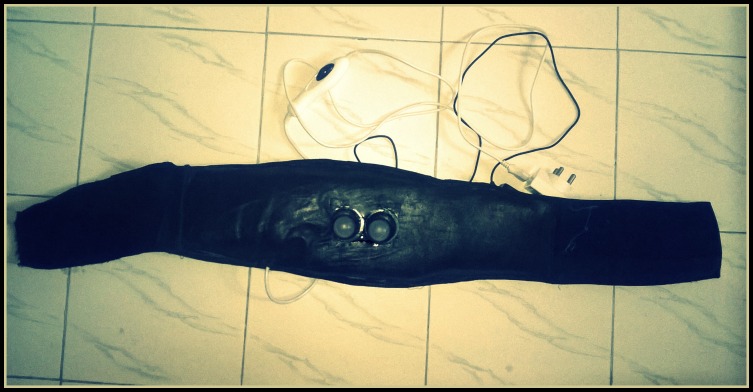
The Comfort-N-Harmony belt - innovative and portable multimodal technology for pain relief

Figure 1 shows the C&H belt in a compact form.

**Figure 2:**
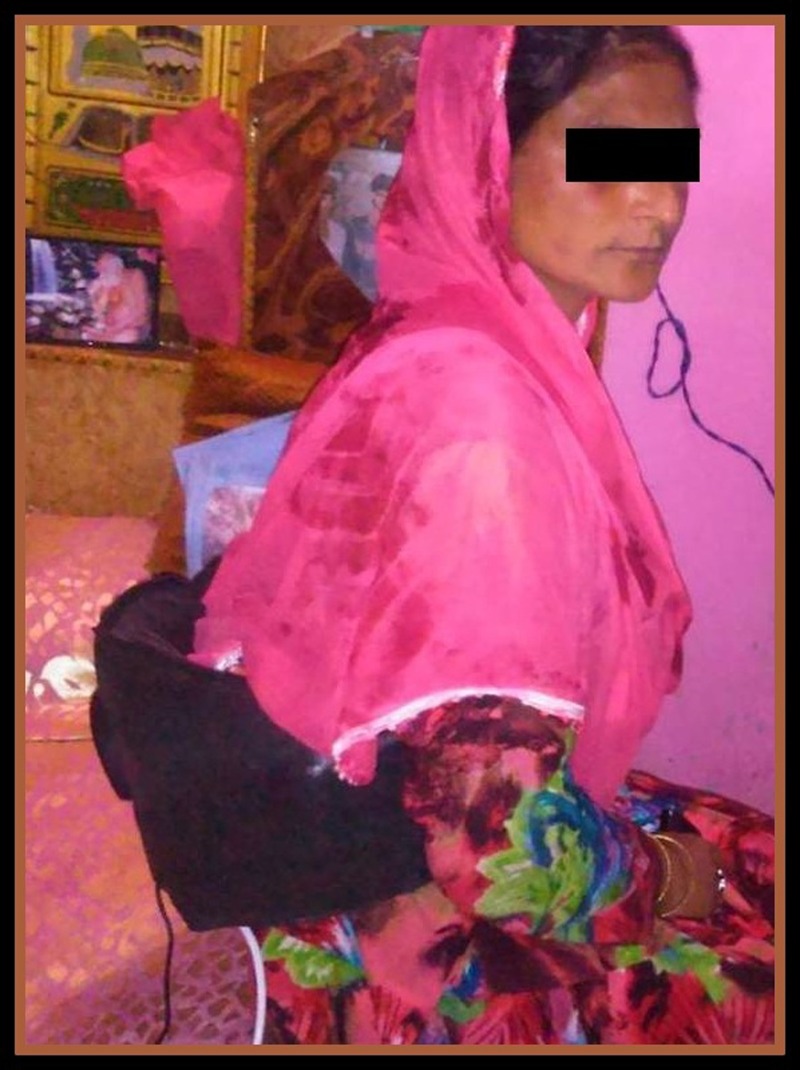
A study participant using Comfort-N-Harmony belt for pain relief

The lumbar belts (C&H belt and plain belt) were used in the respective groups’ volunteers in a double-blind fashion four times a week over a study period of one month. Figure 2 shows a study volunteer (a 35-year-old woman) using our C&H belt at her home for LBP.

### Questionnaires

Before the study was commenced and just after the completion of the one month of study (at the end of one month), all the volunteers were evaluated by the VAS (for pain), the HADS (for anxiety and depression), the RMD (for the function), and the NHP (for quality of life).

### The severity of pain

The severity of pain was measured by the Visual Analogue Scale (VAS). The length of this scale is 10 cm and it bears various descriptors along its length (zero means there is no pain and 10 implies the worst pain). The patient was asked to mark the point on this horizontal line that represents his or her severity of pain. Thus, the distance from the lowest end of this horizontal line up to the point that the patient marked gave the VAS score of severity of pain for that patient.

### The psychological issues

The tool usually used for the assessment of psychological issues associated with pain is the HADS. It enables a researcher to know the risk as well as the level of anxiety and depression, along with the changes in severity of these mental issues. There are 14 items on this scale. While the seven odd number items reflect the anxiety, the rest of seven even number items are the reflectors of depression.

### Function

The Roland-Morris Disability Questionnaire (RMDQ) was used to assess the physical disability in daily activities due to lower back pain. The volunteers were asked to respond to all the items on the questionnaire as a simple yes or no, beginning with the phrase of “because of my low back pain”. One point was given for an answer of “Yes” and zero points for “No”. Thus, a total score for 24 items was calculated. For RMDQ, a higher score means the person is having a greater disability [11].

### Quality of life

To assess the levels of social, emotional, and physical activity of our volunteers for the study, the Nottingham Health Profile (NHP) scale was used. This scale consists of 38 questions. There are six subcategories in this questionnaire, which include the pain, the emotional reaction, sleep, the energy level, physical activity, and the social isolation. For each subgroup, the scores range from 0 (= no problem) to 100 (= all problems listed being present) [12].

### Statistical analysis

For statistical analysis, the SPSS 22.0 software for Windows was used, and p < 0.05 was considered statistically significant. In addition, for demographic and categorical data, the descriptive statistics and Chi-square tests were used. For comparison of results before and after the study period, a paired t- test was used within group evaluation. In order to make a comparison of the intergroup, the student t-test was used. 

## Results

A total of 90 patients (45 in each group), including 46 females (51.11%) and 44 males (48.89%) with a mean age of 46.6 ± 11.23 years and 46.4 ± 10.87 years in the groups A and B, respectively, were included in the study. The mean duration of the symptoms for patients of group A and B was 14.86 ± 2.09 and 14.53 ± 4.88 months, respectively. The sociodemographic characteristics of these patients are shown in Table 1. There were no significant differences in age, gender, educational level and symptom duration between the groups (p < 0.05).

**Table 1:** The Sociodemographic Characteristics of the Study Participants

	C&H Belt, Plus Analgesics Group (Group A)	Placebo Belt, Plus Analgesics Group (Group B)	p-value
Age (years)	46.6 ± 11.23	46.4 ± 10.87	< 0.05
Sex			
Male (n, %)	22 (48.89%)	21 (46.67%)	< 0.05
Female (n, %)	23 (51.11%)	24 (53.33%)	< 0.05
Marital Status			
Single (n, %)	6 (13.33%)	8 (17.78%)	< 0.05
Married (n, %)	39 (86.67%)	37 (82.22%)	< 0.05
Body Mass Index (kg/m^2^)	29.36 ± 3.80	29.84 ± 4.02	< 0.05
Disease Duration (months)	14.86 ± 2.09	14.53 ± 4.88	< 0.05

It was seen that VAS, NHP-sleep, NHP-emotional reaction, and HADS scores were not significantly different between groups (p > 0.05) in the pre-treatment assessments. However, RMDQ, NHP-pain, NHP-physical activity, NHP-energy level, and NHP-social isolation scores were seen to be significantly higher in Group A as compared to the Group B (p < 0.05) as shown in Table 2.

**Table 2:** Comparison of Pre- and Post-Treatment Scores of Pain, Function, Quality of Life, Anxiety, and Depression VAS: Visual Analogue Scale; RMDQ: The Roland Morris Disability Questionnaire; NHP: The Nottingham Health Profile

	C&H Belt, Plus Analgesics Group (Group A)	Placebo Belt, Plus Analgesics Group (Group B)	p-value (Inter-group)
VAS			
Pre-treatment	6.82 ± 2.40	6.65 ± 2.42	> 0.05
Post-treatment	2.76 ± 2.71	3.58 ± 3.64	> 0.05
p-value (Intra-group)	< 0.05	< 0.05	
RMDQ			
Pre-treatment	19.61 ± 5.82	15.25 ± 5.96	< 0.05
Post- treatment	9.2 ± 5.19	9.03 ± 7.02	> 0.05
p-value (Intra-group)	< 0.05	< 0.05	
NHP-Pain			
Pre-treatment	75.13 ± 15.52	53.15 ± 19.45	< 0.05
Post-treatment	35.87 ± 26.51	29.48 ± 27.39	> 0.05
p-value (Intra-group)	< 0.05	< 0.05	
NHP-Physical Activity			
Pre-treatment	65.23 ± 21.76	54.06 ± 20.36	< 0.05
Post-treatment	35.67 ± 19.88	41.36 ± 23.21	> 0.05
p-value (Intra-group)	< 0.05	< 0.05	
NHP-Energy Level			
Pre-treatment	57.50 ± 29.02	36.48 ± 38.88	< 0.05
Post-treatment	42.90 ± 31.29	25.27 ± 30.67	> 0.05
p-value (Intra-group)	< 0.05	> 0.05	
NHP-Sleep			
Pre-treatment	32.30 ± 21.18	23.34 ± 20.15	> 0.05
Post-treatment	27.96 ± 22.86	23.17 ± 24.06	> 0.05
p-value (Intra-group)	> 0.05	> 0.05	
NHP-Social Isolation			
Pre-treatment	31.28 ± 23.82	11.89 ± 22.77	< 0.05
Post-treatment	23.39 ± 27.76	11.05 ± 25.81	> 0.05
p-value (Intra-group)	< 0.05	> 0.05	
NHP-Emotional Reaction			
Pre-treatment	40.78 ± 31.39	26.85 ± 26.71	> 0.05
Post-treatment	32.21 ± 31.40	21.82 ± 23.38	> 0.05
p-value (Intra-group)	> 0.05	> 0.05	
Hospital Depression Scale			
Pre-treatment	7.33 ± 6.12	8.06 ± 6.56	> 0.05
Post-treatment	5.78 ± 5.41	7.25 ± 5.90	> 0.05
p-value (Intra-group)	< 0.05	< 0.05	
Hospital Anxiety Scale			
Pre-treatment	7.26 ± 5.67	9.76 ± 3.98	> 0.05
Post-treatment	6.23 ± 4.23	8.82 ± 3.66	> 0.05
p-value (Intra-group)	< 0.05	< 0.05	

After the study period of one month was over, VAS, RMDQ, NHP-pain, NHP-physical activity and HADS scores in both groups were noted to be significantly improved compared to the pre-treatment scores (p < 0.05). Group A also showed significant improvements in the scores of NHP-energy level and NHP-social isolation (p < 0.05) as shown in Table 2.

Between the two volunteer groups, the post-treatment scores did not significantly show any difference (p > 0.05) (Table 2). Nevertheless, in the comparison of pre- and post-treatment scores, it was seen that the pre-treatment score values of RMDQ, NHP-pain, NHP-physical activity, and NHP-social isolation were much higher in group A compared to the group B, but still these scores were, in a significant way, improved in group A compared to the group B after the study period was over (p < 0.05) as shown in Table 3.

**Table 3:** Comparison of the Differences of Pre- and Post-Treatment Scores Between Groups VAS: Visual Analogue Scale; RMDQ: The Roland Morris Disability Questionnaire; NHP: The Nottingham Health Profile

	C&H Belt, Plus Analgesics Group (Group A)	Placebo Belt, Plus Analgesics Group (Group B)	p-value
VAS	-4.06 ± 2.83	-3.07 ± 2.71	> 0.05
RMDQ	-10.41 ± 6.23	-6.22 ± 5.79	< 0.05
NHP-Pain	-39.26 ± 32.99	-23.67 ± 27.1	< 0.05
NHP-Physical Activity	-29.56 ± 15.28	-12.70 ± 10.39	< 0.05
NHP-Energy Level	-14.60 ± 21.78	-11.21 ± 29.47	> 0.05
NHP-Sleep	-4.34 ± 16.51	-0.17 ± 19.15	> 0.05
NHP-Social Isolation	-7.89 ± 15.67	-0.84 ± 9.59	< 0.05
NHP-Emotional Reaction	-8.57 ± 19.72	-5.03 ± 13.20	> 0.05
Hospital Depression Scale	-1.55 ± 1.28	-0.81 ± 0.83	> 0.05
Hospital Anxiety Scale	-1.03 ± 1.86	-0.94 ± 0.79	> 0.05

## Discussion

The results of the present study comparing the short-term effects of a pain-relief-device based therapy with analgesics versus analgesics with a placebo device on pain, disability, quality of life, and the psychological status of volunteers with chronic LBP demonstrate that both treatment modalities significantly improved pain, disability, two subcategories of quality of life (pain and physical activity), as well as anxiety and depression. The energy level and the social isolation subcategories of quality of life (QOL) were significantly improved in the group A. Also, in group A, although the pre-treatment pain and disability, the physical activity, and the social isolation subscores of quality of life (QOL) were significantly much higher compared to group B, the post-treatment scores were seen to be significantly improved in the group A compared to group B.

Our purpose of inventing this device was:

1. To create a unique pain-relieving device that acts through multiple mechanisms.

2. To make a device as low cost as we could so that it is reproducible anywhere in the world and poor people would get it at affordable prices.

3. To design the device in such a way that its parts are easily replaceable.

4. To make a pain-relieving technology that is entirely different from the devices available today that combines only one or two mechanisms of pain relief.

5. To be able to act for both acute and chronic pain and on almost all body parts (although we performed the present study on the lower back portion only).

As far as this invention of ours is considered, we have scientifically and logically listed the people that can use this pain-relief device based system successfully:

1. People who desire quick pain relief and just cannot wait for the effect of pain medications to start. This device exerts an add-on effect to the effect of the pain medications.

2. Patients who are in chronic pain and have been taking pain medications for a long time but now have become resistant/developed tolerance to those medications.

3. Patients in pain in whom the analgesics should not be used, like for example – the patients who have peptic ulcers due to the chronic use of analgesics, patients that are using multiple medications for some other disease or diseases.

4. Patients in pain who simply refuse to take medications as they live with a belief that medications make them worse and they often request prescriptions like bed rest and hot water bottles.

5. Non-compliant patients (who don't take their prescribed pain medications on time or who often forget to do so).

6. Patients who want to have quick relief of muscular pain that aroused as a hectic and excessive workload during the day. 

7. Patients who are fasting for religious purposes and do not want to break their fast by taking an analgesic by mouth.

In a study conducted by Roland Staud, et. al., 28 pain-free normal controls, 29 patients suffering from fibromyalgia, and 19 individuals with back (or neck pain) were included. It was seen that the application of homotopic vibrotactile stimulation to the concerned areas resulted in 40% reductions in pain in all the subject groups [13]. Vibration induced analgesia has been related in various studies to A-beta mediated afferent inhibition of the dorsal horn nociceptive neurons [14, 15]. In our study, the device which we used had 3 vibrators placed at different positions providing vibro-tactic stimuli at different frequencies and intensities as adjusted by the study participant.

Using heat in the form of head pads, hot water bottles has been an age-old therapy for managing musculoskeletal pain. This is scientifically proven mechanism too and research is being conducted to treat pain via infra-red devices. Even a Cochrane review has cited moderate evidence in support of the analgesic effect of using superficial heat therapy in patients with acute or sub-acute low back pain [16].

Heat therapy for chronic back pain has been proven in a double-blind, placebo-controlled trial to be easy to use, effective and safe in pain reduction by 50% over 6 weeks [17]. Our device had a heat element within a heat pad which generated therapeutic amount of heat for treating LBP, although we used it in our volunteers for a study period of 4 weeks (1 month) only instead of the 6 weeks used in the latter study. Most of the participants in our study also revealed that they felt a relaxing effect of heat when used alternately with cold (which they reported being soothing) at five-minute intervals each.

Menthol, after topical application, causes a feeling of coolness due to stimulation of 'cold' receptors by inhibiting calcium currents of neuronal membranes. Since calcium channel blockers are endowed with analgesic properties, the potential antinociceptive effect of menthol has been proved in many studies [18]. Peppermint is the natural cooling oil whose product menthol is widely employed in topical pain medications. Menthol was hypothesized to induce the cooling sensation by activating TRPM8, an ion channel in present in cold-sensitive sensory neurons. Various studies have been able to identify more targets of menthol, which include the TRPA1, an irritant receptor, and also some neurotransmitter receptors and voltage-gated ion channels. It used to be a debatable topic that which of these targets are actually associated with the menthol-induced pain relief, or to the irritating effects that are associated with therapy with menthol. In a study conducted by Liu B, et al., it was proven that TRPM8 is the principal mediator of menthol-induced pain relief of acute as well as inflammatory types of pain [19]. We used menthol in the form of a natural peppermint oil in one of the roll-on bottles of the device, which were directed at the pain site and controlled by the vibrations of the vibrator(s).

Camphor is a well-known counter-irritant and available naturally and employed in various liniments and balms available as topical analgesics. It is one of the oldest used medical product for pain relief, but it has not completely come to light that how it works on a molecular level to relieve the pain. Some researchers have hypothesized that TRPV1 desensitization contributes to the pain-relieving actions of another topical analgesic capsaicin. Even though camphor is known to activate TRPV1 less effectively, it was found in a study that camphor application caused desensitization of TRPV1 in a quicker and more complete way than capsaicin. In the same study, it was also found to cause inhibition of many other related TRP channels, like ankyrin-repeat TRP 1 (TRPA1). So, it is now believed that camphor-induced blockade of TRPA1, as well as desensitization of TRPV1, underlies the pain relieving effects of camphor [20]. We used camphor in another roll-on bottle at the pain site fixed in the device which was also controlled by the vibrations of the vibrator(s).

Various lumbar belts are employed in ergonomics in order to prevent the low back injury, while lumbar orthoses are being used in the clinical settings for a wide range of conservative as well as postsurgical LBP management. These two devices function in with a similar biomechanical mechanism of action by not only reducing the range of motion of the trunk but also by increasing the overall stiffness of the trunk. There is sufficient evidence that the people who wear these devices have claimed to feel "more stable" and "safer" while they are doing physical work. Lumbar spinal orthoses have achieved considerable fame in many self-reported survey studies, in which people have claimed that using these orthoses helped them continue their daily activities with considerable minimal discomfort [21]. Our device helped people in improving their quality of life by allowing them to do their work very easily while using the belt.

Using essential oils in therapeutic formulations have been linked to central analgesic effects. These fragrant oils like lemon oil have been shown in various studies to increase the production of endorphins which cause a decrease in the sensation of pain and provide a feeling of well-being [22, 23]. We used a strongly fragrant essential oil (lemon oil) as an adjuvant to camphor and peppermint in their respective roll-on bottles to add to their own typical essential oil odors. In a separate survey, 97.78% of our volunteers claimed that they liked the smell of the essential oils and gave them an internal feeling of wellbeing.

Finally, a non-invasive, non-drug, non-pain, non-contact, non-addictive method of pain relief is being developed which was used in ancient times but later disproved due to less evidence of proposed mechanisms of action. Some studies have demonstrated a little degree of analgesia although the absence of analgesic effect has not been clearly proven yet. A magnet arrangement recipe has been proposed by the authors of a study in which they achieved a pain relief effect of more than 80% in animal experiments in the writhing test. As a matter of fact, the writhing test is a well known screening method for pain in animals as well as predictor of human experimental results [24]. We placed numerous magnets scattered in the device which doesn't interfere with the working of the device and at the same time perform their own work of potential pain relief.

To our knowledge, this study is the first one to compare the effectiveness of using such a multi-modal pain relief portable device based system plus analgesic intake, with analgesics intake plus placebo device (to remove the bias within the groups) in chronic LBP. This is also the first such study from Southeast Asia. Also, our medical literature lacks sufficient data on this burning issue.

The major limitation of the present study is that it registered only short-term outcomes. Nevertheless, this study may have many significant implications in medical field, because it has evaluated the effectiveness of an innovative method of combining all the possible effective complementary methods of pain relief which may help relieve the burden of LBP patients we are experiencing today.

## Conclusions

We have laid the foundation stone of an innovative multimodal method of pain relief. As research on multimodal devices/robots for pain relief is in its early stages of development, further collaborative research is needed to develop a more solid understanding concerning the nature of analgesia and how device-based systems can best be used for long-term pain relief. Since the time of heavy and judicious use of robotics in medical science is quite near, future research should also continue to explore an answer to the question – Can we replace analgesics with mini portable robotic devices for pain relief? This system will allow physicians, neurologists, and orthopedists to have more time to figure out and eliminate the root cause of pain in the analgesics-refractory/tolerant patients while using the portable device-based systems for pain relief in such patients.

## References

[1] Baron R, Binder A, Attal N, Casale R, Dickenson AH, Treede RD (2016). Neuropathic low back pain in clinical practice. Eur J Pain.

[2] Meucci RD, Fassa AG, Faria NM (2015). Prevalence of chronic low back pain: systematic review. Rev Saude Publica.

[3] Ghildayal N, Johnson PJ, Evans RL, Kreitzer MJ (2016). Complementary and alternative medicine use in the US adult low back pain population. Glob Adv Health Med.

[4] Chung SH, Her JG, Ko T, Ko J, Kim H, Lee JS, Woo JH (2013). Work-related musculoskeletal disorders among Korean physical therapists. J Phys Ther Sci.

[5] Heneweer H, Staes F, Aufdemkampe G, van Rijn M, Vanhees L (2011). Physical activity and low back pain: a systematic review of recent literature. Eur Spine J.

[6] Hooten WM, Cohen SP (2015). Evaluation and treatment of low back pain: A clinically focused review for primary care specialists. Mayo Clin Proc.

[7] Sherman KJ, Cherkin DC, Connelly MT, Erro J, Savetsky JB, Davis RB, Eisenberg DM (2004). Complementary and alternative medical therapies for chronic low back pain: What treatments are patients willing to try?. BMC Complement Altern Med.

[8] van Middelkoop M, Rubinstein SM, Kuijpers T, Verhagen AP, Ostelo R, Koes BW, van Tulder MW (2011). A systematic review on the effectiveness of physical and rehabilitation interventions for chronic non-specific low back pain. Eur Spine J.

[9] van Tulder MW, Furlan AD, Gagnier JJ (2005). Complementary and alternative therapies for low back pain. Best Pract Res Clin Rheumatol.

[10] Kanodia AK, Legedza AT, Davis RB, Eisenberg DM, Phillips RS (2010). Perceived benefit of complementary and alternative medicine (CAM) for back pain: A national survey. J Am Board Fam Med.

[11] Roland M, Morris R (1983). A study of the natural history of back pain: Part I: Development of a reliable and sensitive measure of disability in low-back pain. Spine (Phila Pa 1976).

[12] Hunt SM, McKenna SP, McEwen J, Williams J, Papp E (1981). The Nottingham Health Profile: subjective health status and medical consultations. Soc Sci Med A.

[13] Staud R, Robinson ME, Goldman CT, Price DD (2011). Attenuation of experimental pain by vibro-tactile stimulation in patients with chronic local or widespread musculoskeletal pain. Eur J Pain.

[14] Salter MW, Henry JL (1990). Differential responses of nociceptive vs. non-nociceptive spinal dorsal horn neurones to cutaneously applied vibration in the cat. Pain.

[15] Salter MW, Henry JL (1990). Physiological characteristics of responses of wide dynamic range spinal neurones to cutaneously applied vibration in the cat. Brain Res.

[16] French SD, Cameron M, Walker BF, Reggars JW, Esterman AJ (2006). A Cochrane review of superficial heat or cold for low back pain. Spine (Phila Pa 1976).

[17] Gale GD, Rothbart PJ, Li Y (2006). Infrared therapy for chronic low back pain: a randomized, controlled trial. Pain Res Manag.

[18] Galeotti N, Di Cesare Mannelli L, Mazzanti G, Bartolini A, Ghelardini C (2002). Menthol: a natural analgesic compound. Neurosci Lett.

[19] Liu B, Fan L, Balakrishna S, Sui A, Morris JB, Jordt SE (2013). TRPM8 is the principal mediator of menthol-induced analgesia of acute and inflammatory pain. Pain.

[20] Xu H, Blair NT, Clapham DE (2005). Camphor activates and strongly desensitizes the transient receptor potential vanilloid subtype 1 channel in a vanilloid-independent mechanism. J Neurosci.

[21] Cholewicki J, McGill KC, Shah KR, Lee AS (2010). The effects of a three-week use of lumbosacral orthoses on trunk muscle activity and on the muscular response to trunk perturbations. BMC Musculoskelet Disord.

[22] Ceccarelli I, Lariviere WR, Fiorenzani P, Sacerdote P, Aloisi AM (2004). Effects of long-term exposure of lemon essential oil odor on behavioral, hormonal and neuronal parameters in male and female rats. Brain Res.

[23] Nie H, Shen YJ (2002). Effect of essential oil of Radix Angelicae Dahuricae on beta-endorphin, ACTH, NO and proopiomelanocortin of pain model rats (Article in Chinese). Zhongguo Zhong Yao Za Zhi.

[24] László J, Reiczigel J, Székely L, Gasparics A, Bogár I, Bors L, Rácz B, Gyires K (2007). Optimization of static magnetic field parameters improves analgesic effect in mice. Bioelectromagnetics.

